# Colloidal metasurfaces displaying near-ideal and tunable light absorbance in the infrared

**DOI:** 10.1038/ncomms8325

**Published:** 2015-06-23

**Authors:** Matthew J. Rozin, David A. Rosen, Tyler J. Dill, Andrea R. Tao

**Affiliations:** 1 NanoEngineering Department, University of California, San Diego, 9500 Gilman Drive MC 0448, La Jolla, California 92093-0448, USA

## Abstract

Metasurfaces are ultrathin, two-dimensional arrays of subwavelength resonators that have been demonstrated to control the flow of light in ways that are otherwise unattainable with natural materials. These arrays are typically composed of metallic Ag or Au nanostructures shaped like split rings, nanowire pairs or nanorods (commonly referred to as meta-atoms) that are arranged to produce a collective optical response spanning an impressive range of properties, from the perfect absorption of incident light to superresolution imaging. However, metasurfaces pose major challenges in their fabrication over large areas, which can be prohibitively expensive and time consuming using conventional nanolithography techniques. Here we show that differently shaped colloidal nanocrystals can be organized into metasurface architectures using robust, scalable assembly methods. These metasurfaces exhibit extreme in-plane electromagnetic coupling that is strongly dependent on nanocrystal size, shape and spacing. Colloidal metasurfaces that display near-ideal electromagnetic absorbance can be tuned from the visible into the mid-infrared wavelengths.

## Abstract (web summary)

Metasurfaces are arrays of subwavelength structures that are tailored to produce specific optical responses. Rozin *et al.* show that large-area metasurfaces can be readily fabricated by self-assembly of colloidal nanocrystals of different geometries, producing tunable reflectance and absorbance properties.

## Graphical abstract

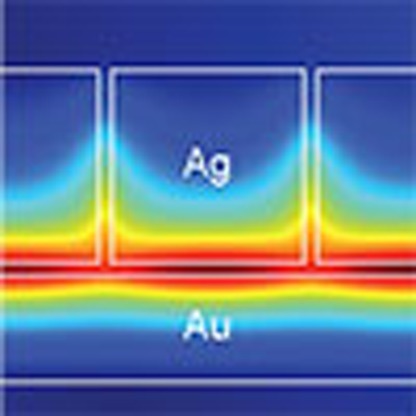

Colloidal nanocrystals have been successfully demonstrated as nanoscale building blocks for the assembly of large-area plasmonic metamaterials^1^,^2^,^3^. Metal nanocrystals serve as plasmonic meta-atoms by supporting the excitation of localized surface plasmon resonances (LSPRs), where light impinging on a metal nanostructure couples to the free electrons in the metal^4^,^5^. To form a metasurface, nanocrystals must be organized into macroscale arrays that produce the optical response of an effective medium^6^,^7^,^8^. Changing the size and shape of the individual nanostructures, as well as changing the arrangement and spacing within the array enables tuning of the permittivity and permeability of the metasurface without changing the dielectric environment^9^,^10^. In demonstration of such an approach, Moreau *et al.*^11^ recently fabricated a near-perfect electromagnetic absorber composed of colloidal Ag nanocubes deposited onto an Au thin film. This sandwich structure produces regions of intense light confinement in the gaps between the nanocube and the Au surface. Absorbance in the visible range is tuned by controlling the dielectric spacing between these two components. To achieve metasurface operation extending into the infrared wavelengths, meta-atoms that support LSPR excitation at longer wavelengths can be chosen^12^. However, this approach is limited by the ability to chemically synthesize nanocrystals within a specified size or shape range.

An alternative strategy to tailor the wavelength range and bandwidth of a colloidal metasurface is to exploit electromagnetic coupling between individual meta-atoms. Typically, when metallic nanoparticles are placed in proximity to each other, inductive or capacitive interaction between the nanoparticles will give rise to a coupled electromagnetic response^13^. Thus, in-plane coupling between meta-atoms is expected to provide an additional design variable for colloidal metasurfaces. Here we exploit self-assembly as a powerful fabrication approach to overcome limitations in scalability, tunability and design of colloidal metasurfaces. We examine the optical response of metasurfaces comprised of colloidal Ag nanocubes with interparticle spacings in the range of a 2–100 nm. In the case of Ag nanocubes on a metal film (abbreviated here as NOM), in-plane coupling between neighbouring nanocubes gives rise to give tunable reflectance and absorbance properties over a wide wavelength range, out to 3 μm.

## Results and Discussion

**Figure 1:**
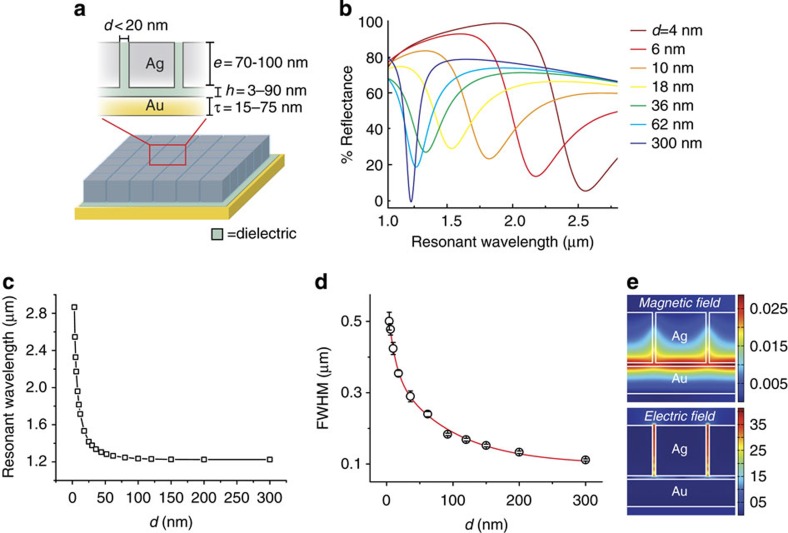
NOM metasurface geometry and simulated optical response for varying interparticle spacing. (**a**) Schematic of the model NOM structure with Ag nanocubes assembled atop an Au film with a dielectric spacer. Adjustable parameters include interparticle distance (*d*), length of nanocube edge (*e*), thickness of Au film (*τ*) and dielectric spacer height (*h*). (**b**) Reflectance spectra of simulated NOM arrays of 92 nm Ag nanocubes with *d*=4–300 nm. (**c**) Plot showing exponential trend of fundamental resonance wavelength as a function of interparticle spacing. (**d**) Plot of the FWHM of the fundamental resonance for decreasing *d*. (**e**) Magnetic and electric field intensities calculated at the fundamental resonance wavelength (*λ*=2.54 μm) for a close-packed NOM metasurface with *d*=4 nm. The high-field regions (red) for the H-field are supported below the nanocube, whereas they are apparent in the inter-nanocube gap for the E-field.

Previously, NOM metasurfaces with Ag nanocube surface coverages between 4 and 17% were examined^11^. These surface coverages are correlated to interparticle spacings of 190–350 nm where interactions between neighbouring nanocubes are negligible^14^. We examined the optical response for NOM metasurfaces composed of Ag nanocubes (with edge length, *e*=92 nm) that possess different interparticle spacings, *d*, between 3 and 300 nm using two-dimensional (2D) finite-difference time-domain (FDTD) simulations. (Fig. 1) The fundamental mode, defined as the lowest-order resonance of the metasurface, is exhibited by a sharp decrease in the reflectance spectra corresponding to near-perfect absorption (Fig. 1b). The resonant wavelength of the fundamental mode shows an exponential decrease when plotted as a function of *d* (Fig. 1c). This relationship has been well-observed for plasmonic nanoparticle pairs^15^, clusters^16^, and arrays^17^. For spacings of *d*=3 nm, nanocubes experience strong electromagnetic coupling and the fundamental mode is centred at *λ*=2.87 μm. As *d* is increased, this value approaches the resonance wavelength reported for a well-spaced nanowire array^18^, with *λ*=1.225 μm for *d*=300 nm. These large interparticle spacings result in an array of meta-atoms in the weak-coupling limit, where the optical resonance resembles that of an isolated particle.

Figure 1d plots the linewidth of the fundamental mode as a function of nanocube spacing. As *d* is increased, the linewidth sharpens significantly from 0.50 μm for *d*=4 nm to 0.11 μm for *d*=300 nm. The NOM structure exhibits significantly different behaviour from electromagnetically coupled nanoparticle pairs, where the linewidth for particles spaced by <20 nm decreases to <50% of the linewidth observed for isolated particles^19^. This difference in linewidth behaviour results from the occurrence of both out-of-plane and in-plane coupling in the NOM metasurface. Out-of-plane coupling refers to nanoparticle–surface interactions between the nanocube and the underlying metallic film. Nanoparticle–surface interactions typically result in a red shift of the dipolar LSPR without significant peak broadening^20^ (see Supplementary Fig. 1). In-plane coupling refers to interactions between neighbouring nanocubes. Increasing the density of nanocubes, and thus decreasing *d* of the NOM structure, results in a quasi-continuous resonance from the coupled LSPRs of neighbouring nanocubes. Out-of-plane coupling between this quasi-continuous LSPR resonance and the metal film has the effect of markedly broadening (due to radiation damping) and red shifting the fundamental mode of the NOM structure. This is illustrated in Fig. 1e, which shows the simulated magnetic and electric field current densities for the fundamental mode (*λ*=2.54 μm) for the close-packed nanocube NOM structure. A quasi-continuous gap mode between the nanocubes and the metal film effectively extends across the entire metasurface. An electric field node is located in the region of greatest intensity for the corresponding magnetic field, and the region of greatest electric field intensity is located within the interstitial spaces between neighbouring nanocubes. The local field volume is spread over multiple nanocubes, and for an idealized symmetric geometry, increasing the fundamental gap mode volume by ∼2.8 times in comparison with isolated nanocubes with *d*>300 nm (See Supplementary Note 1).

**Figure 2:**
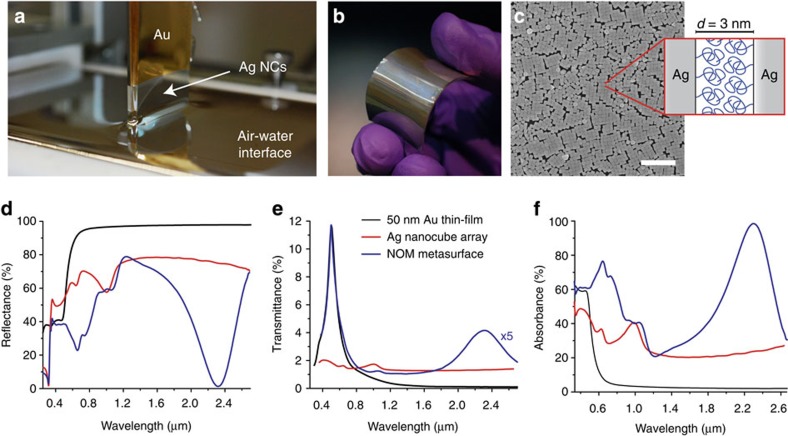
Fabrication and optical response of a NOM metasurface. (**a**) Image of the deposition process after Ag nanocube array assembly at an air–water interface. (**b**) Demonstration of metasurface fabrication onto a large-area, flexible elastomer substrate. (**c**) Scanning electron microscope image showing close-packed Ag nanocubes after deposition, with a measured spacing of 3 nm, which occur due to polymer grafts at the Ag surface. Scale bar=1.0 μm. (**d**–**f**) Reflectance, transmittance, and absorbance spectra of a close-packed NOM metasurface using Ag nanocubes with *e*=92 nm, with 98% absorbance at the fundamental resonance (blue curve). For comparison, spectra for an array of close-packed Ag nanocubes (red), and a bare Au thin film (black) are shown.

Figure 2 shows the fabrication and resulting optical properties of a close-packed NOM metasurface. We employ a Langmuir–Blodgett trough to form close-packed Ag nanocube arrays^17^ at an air–water interface (Fig. 2a) that can then be transferred onto arbitrary substrates, including flexible and non-planar supports (Fig. 2b). Ag nanocubes were synthesized using previously reported colloidal methods^21^,^22^ and then compressed into a dense monolayer as shown in the scanning electron microscope (SEM) image in Fig. 2c. Interparticle spacing within the regions of the monolayer that display domains with close packed ‘face-to-face' nanocubes is controlled by the presence of long polymer grafts that passivate the nanocube surface, yielding separation distances of approximately 2–3 nm, as measured by transmission electron microscopy (Supplementary Fig. 2). The nanocube monolayer is then transferred onto a solid support using a mechanized dip coater. The solid support is composed of a 50 nm Au thin-film sputtered onto soda-lime glass. The Au thin–film is passivated with a molecular monolayer of alkanethiols, which serves as a dielectric spacer ∼3 nm thick.

Figure 2d,e shows the near-normal (*θ*=4°) reflectance and normal transmittance spectra of a close-packed NOM metasurface composed of Ag nanocubes (*e*=92 nm). The fundamental mode is clearly observed at *λ*=2.32 μm, where the reflectance spectrum exhibits a sharp decrease to a minimum per cent intensity of 1.3%, correlating to an absorbance of 98% (Fig. 2f). In comparison, a pristine Au thin film has a flat reflectance response of ∼97% in the near-infrared range and a stand-alone Ag nanocube array has a reflectance intensity within a range of 71–78% at similar wavelengths. The drastic reduction in reflectance for the NOM metasurface can only be attributed to electromagnetic coupling of the nanocube array with the Au thin film. The per cent transmittance of the NOM metasurface (Fig. 2e) is a maximum of 0.83% at the fundamental mode wavelength. Transmittance can be further decreased by utilizing a thicker Au film as the solid support (see Supplementary Fig. 3). It is of particular note that the entire thickness for this metasurface is ∼150 nm—over 16 times less than the resonant wavelength of the NOM metasurface.

**Figure 3:**
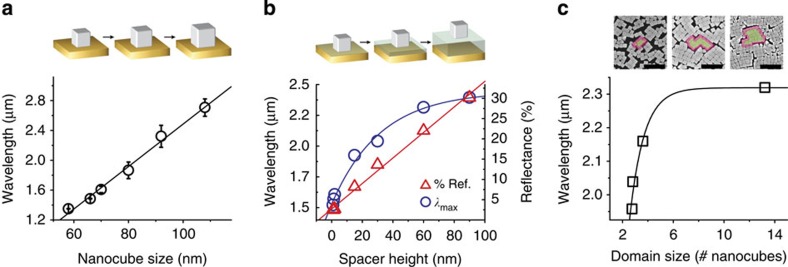
Tunable parameters of experimental NOM metasurfaces (**a**) NOM metasurfaces with various sized nanocubes ranging from *e*=58–108 nm. The linear trend of the fundamental resonance wavelength as a function of nanocube size is shown (black line). Error bars display the s.d. of the resonance wavelength position between multiple NOM metasurfaces fabricated from nanocubes with the same average size. (**b**) NOM metasurfaces with increasing dielectric spacer heights from a few nanometres to *h*=90 nm. As *h* is increased, the quality factor of the resonant gap is reduced, represented by the linear increase in minimum reflectance at the fundamental mode with increasing spacer height (red line). The corresponding resonant wavelength of the gap mode is shown for each metasuraface at variable spacer height (blue line). (**c**) Representative SEM images and wavelength dependence of metasurfaces with varying nanocube domain sizes (black line). Scale bars=500 nm.

The advantage of using this self-assembly technique is the ability to tune the resonant wavelength of the NOM metasurface using a wide range of experimental design parameters. Fig. 3 shows metasurface architectures where the fundamental mode is tuned between 1.34 and 2.72 μm by changing Ag nanocube size, dielectric spacer height or nanocube packing density and order. These results lead to some general rules regarding the performance of these self-assembled metasurfaces:

First, the wavelength of the fundamental mode increases linearly with nanocube size, owing to the linear increase in dipolar resonance wavelength for non-coupling colloidal nanocubes of increasing size (Supplementary Fig. 4). Figure 3a shows the effect of varying nanocube size on the position of the NOM fundamental resonance. The resonance position in the absorbance spectra is plotted for metasurfaces with Ag nanocubes of different sizes (*e*=59±4, 70±4, 80±8, 92±5 and 108±6 nm). The corresponding reflectance, transmittance and absorbance spectra are shown in Supplementary Fig. 5. Variations in spectral lineshape are primarily attributed to heterogeneities in size and shape within each colloidal nanocube dispersion, as summarized in Supplementary Table 1. Second, increasing Ag nanocube size decreases absorption efficiency of the fundamental mode. This is likely due to the increase in scattering cross-section observed for larger metal particles^23^. The NOM metasurfaces that yielded the highest reduction in reflectance were the films with *e*=70 nm and 92 nm nanocubes, with reflectance minima of 2.86% at 1.57 μm and 2.13% at 2.56 μm, respectively. Using 2D FDTD simulations, we model the optical response for close-packed metasurfaces built with uniform Ag nanocubes ranging in size from 50 to 200 nm, while holding all other design parameters constant (see Supplementary Fig. 6). To extend the perfect-absorbing fundamental mode out to *λ*=3.0, 4.0, and 5.0 μm, NOM metasurfaces would require nanocubes with edge lengths *e*=108.1, 143.7, and 179.3 nm, respectively.

For close-packed NOM metasurfaces, increasing gap height *h* weakens out-of-plane field confinement between the nanocubes and the Au film and increases in-plane field confinement between nanocubes. This results in a red shift of the fundamental mode wavelength. Figure 3b plots the effect of varying dielectric spacer height from 3 to 90 nm for NOM metasurfaces (*e*=70 nm). To achieve small *h* between 2.9 and 4.0 nm we varied the alkyl chain-length of the molecular monolayer covering the Au thin film. For *h* between 15 and 90 nm, the height of the dielectric spacer was varied by spin-coating thin films of poly(methyl methacrylate) (PMMA) onto the Au film before nanocube deposition. Intuitively, an increase in *h* should reduce the quality factor of the resonant gap. We observe a sevenfold increase in per cent transmission for NOM metasurface fabricated with these polymer dielectric spacers, but in each case the transmittance remains <2% (see Supplementary Fig. 7). We also observe that the resonant wavelength of the fundamental gap mode red shifts with increasing *h*, from *λ*=1.52 μm at *h*=3 nm to *λ*=2.46 μm at *h*=90 nm. This significant red shift is due to the increasing volume of the fundamental gap mode. This is in direct contrast to what is observed for metasurfaces composed of isolated metallic nanostructures, where, for small *h* (often *h*<50 nm), the fundamental mode wavelength blue shifts with increasing *h* (refs 20, 24, 25). For large values of *h* (where *h*≈*e*) we still observe a substantial suppression of reflectance at the fundamental resonance of the metasurface. This is advantageous because various dielectric materials (for example, polymers and active materials) can be incorporated into the spacer layer without sacrificing the performance of the metasurface absorber.

We observe that the overall order of the self-assembled metasurface plays a key role in determining its optical function. Figure 3c shows SEM images of three representative NOM metasurfaces, clearly showing the non-uniform spacing and arrangement of nanocubes on the metasurface. We observe two distinct populations of inter-nanoparticle spacings: close-packed nanocubes within ordered domains (*d*≈2–12 nm); and well-spaced nanocubes (*d*>100 nm) located at the edges of neighbouring domains. The presence of these two spacing regimes over the long range order of the metasurface can be clearly seen in Supplementary Fig. 8. Because electromagnetic coupling increases exponentially with decreasing distance (Fig. 1c), the strong coupling between close-packed nanocubes is the primary determinant of the fundamental resonant wavelength of the metasurface. Arrays with larger domain sizes possess better order, given that grain boundaries present defects in the array where the nanocubes are not close-packed. In the extreme limits of order and disorder, a NOM metasurface would possess an infinite domain size or a domain size of one nanocube, respectively. We have observed that the overall order of the nanocube array is likely determined by the size and shape distribution of the colloidal nanocubes; while surface chemistry also plays a role (since it dictates interparticle spacing and interactions at the air–water interface), we did not observe that the polymer ligands grafted to the nanocubes were critical in affecting domain size. Figure 3c shows the resonant position of the fundamental mode for the NOM metasurfaces displayed in the SEM images, with the corresponding reflectance spectrum shown in Supplementary Fig. 9. As the film becomes more ordered, the wavelength of the fundamental mode asymptotically approaches the wavelength predicted for a perfect array (Fig. 1b). This red shift is attributed to a decrease in the average interparticle distance between nanocubes, consistent with our simulations. We also observe that an increase in domain size results in an increase in optical absorbance at the fundamental mode. Disorder within the NOM array increases the instances of isolated or poorly coupled nanocubes, contributing to radiation loss via scattering and the creation of a ‘leaky' fundamental gap mode.

**Figure 4:**
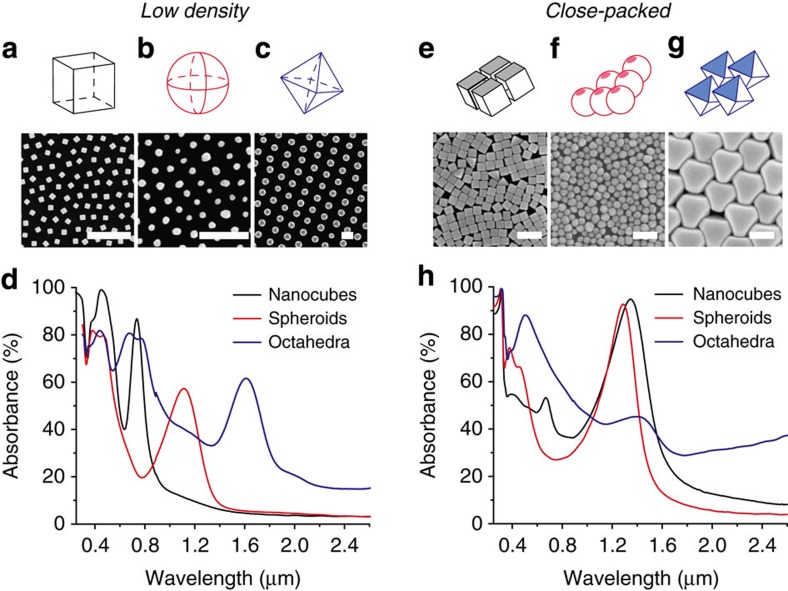
Effect of Ag nanocrystal packing density and shape on gap-mode cavity. (**a**–**c**) SEM images of well-spaced arrays of Ag cubes, spheroids and octahedra; scale bars=500 nm. Above each SEM image is a geometric representation of the individual nanocrystal used in each metasurface. (**d**) Experimental absorbance spectra for metasurface arrays with well-spaced nanocrystals of various shapes. (**e**–**g**) SEM images of close-packed metasurfaces; scale bars=200 nm, with above diagram showing packing structure for each corresponding nanocrystal shape. (**h**) Experimental absorbance spectra corresponding to metasurface arrays with close-packed nanocrystals of various shapes.

Finally, we demonstrate that these self-assembled metasurfaces can be achieved with a number of differently shaped meta-atoms. Figure 4 compares the optical resonances of metasurfaces composed of Ag nanocrystals with the following shapes and sizes: cubes (59±4 nm); spheroids (65±11 nm); and octahedra (235±12 nm). We compare the optical absorbance spectra for these metasurfaces fabricated at low (Fig. 4a–d) and close-packed (Fig. 4e–h) nanocrystal densities. The low density metasurfaces were fabricated by depositing nanocrystal arrays with a surface coverage of ∼20%, corresponding to arrays where the nanocrystals are spaced by at least two times their effective particle diameter. Close-packed metasurfaces were fabricated with nanocrystals separated by *d*<4 nm. Near-normal incidence reflectance and transmittance spectra were collected for each metasurface, shown in Supplementary Figs 10 and 11. Figure 4d,h shows the absorbance spectra obtained by subtracting the measured reflectance and transmittance values from 100%. For the low-density metasurfaces, the dominant peak observed in each of the absorbance spectra correspond to the fundamental optical resonance generated within the cavity between the nanocrystal and the metal thin film. The wavelength of the fundamental mode scales with the size of the meta-atom, consistent with previous studies^26^. Per cent absorbance is highest for meta-atoms that possess flat facets that lie parallel to the underlying metal film.

To compare the optical performance of each meta-atom, we use the following expression to calculate quality factor for the fundamental gap mode: *Q*=*λ*_*r*_/*δλ*, where *λ*_*r*_ is the resonant wavelength and *δλ* is the FWHM obtained from the absorbance spectrum. While there are numerous factors that contribute to the cavity quality of complex systems including local geometry parameters, refractive index, and absorbance coefficients^27^, this serves as a simple comparison of our three differently shaped meta-atoms. The quality factors of the three meta-atoms are described by the relation in equation (1):


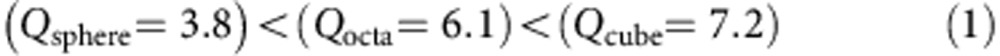


This order is attributed to the shape of the nanocrystals. Both the octahedra and the nanocubes possess atomically smooth facets^16^ that form a neat parallel-plate cavity with the underlying Au thin film. Such cavities support high-quality optical resonances resulting from low-loss confinement between the nanocrystal and the Au film. The shape of the octahedra is such that a significant portion of the nanocrystal extends out beyond the footprint of the nanocrystal-film cavity, resulting in a lower *Q* than the nanocubes. Electromagnetic coupling between spheroids and the Au film produces gap modes where the field is poorly confined to the nanocrystal-film cavity due to the high curvature of the nanocrystal, resulting in the lowest *Q* of the three shapes.

For the close-packed metasurfaces, we expect that strong coupling between the nanocrystal arrays at the Au thin film will produce a large red shift for the wavelength of the fundamental gap mode. This is the case for both nanocubes and octahedra. For the octahedra, this redshift is so large that the resonant wavelength of the fundamental mode is beyond the scope of our detector, past 3 μm. Electrodynamic simulations confirm that the close-packed octahedra metasurface possesses a fundamental mode near *λ*=3.60 μm. We attribute the absorbance peak located at *λ*=1.40 μm to the second-order mode of the metasurface. In contrast, close-packed Ag spheroids exhibit only a small redshift by comparison (*Δλ*=0.18 μm), with a resonant wavelength of *λ*=1.29 μm for the fundamental mode. While spherical nanoparticles are known to experience strong plasmonic coupling when close packed^28^, this result shows that there is little interaction between the in-plane (that is, interparticle) and the out-of-plane (that is, particle-film) gap modes. This is further confirmed by characterization of spheroid metasurfaces that possess varying nanocrystal surface coverage (see Supplementary Fig. 12). An increase in surface coverage increases the total per cent absorbance of the metasurface with little change to the *Q* of the fundamental mode, indicating that Ag spheroids behave like minimally interacting optical resonators. This comparison between spheroids, cubes and octahedra further highlights the importance of meta-atom shape on the ability to tune metasurface resonances into the infrared wavelengths.

Our work demonstrates that metasurfaces enabled by self-assembly can be fabricated in a scalable, robust and tunable manner. This work paves the way for such advances in metamaterials development, and specifically in the demonstration of large-area assembly techniques based on dip coating. We expand the active optical range of colloidal metasurfaces from the visible to the mid-IR wavelengths by utilizing in-plane light confinement between meta-atoms and by tuning meta-atom parameters such as size, shape and arrangement. These colloidal metasurfaces exhibit extreme light confinement, with subwavelength optical cavities that possess dimensions <200 nm and operating wavelengths of a few microns. While these colloidal metasurfaces exhibit extreme light confinement, our highest performing metasurfaces are less than perfect and exhibit 98% absorbance at the fundamental resonance wavelength. In addition, assembly defects likely play a major role in limiting the uniformity and bandwidth of metasurface performance. Future work will examine the defect tolerance and the possibility of defect engineering for these colloidal metasurfaces, and may provide new methods for designing the structure and function of metamaterials architectures.

## Methods

### Ag nanocube preparation

Ag Nanocubes were synthesized via a polyol method published elsewhere^22^. In brief, AgNO_3_ is reduced in a solution of pentanediol, CuCl_2_, and polyvinylpyrrolidone (PVP) (*M*_w_=55,000). PVP serves as a selective capping agent that controls nanocube nucleation and growth. The reaction was allowed to proceed until the resulting colloidal dispersion turned an opaque yellow-green colour. To remove excess reactants, the nanocube dispersion product was centrifuged (2,700 r.p.m. for 10 min) using a Thermo Scientific CL2 Centrifuge, and the resulting precipitate was redispersed and diluted in an ethanol and water mixture, and then vacuum filtered (Millipore Durapore membranes, with 0.65 and 0.45 μm, then 0.22 μm pore sizes) to remove any larger, unwanted particles. To prepare the Ag nanocubes for Langmuir–Blodgett film deposition, this dispersion is further concentrated by centrifugation (3,400 r.p.m. for 20 min) and the precipitate is redispersed in ethanol. This process was repeated three times before finally dispersing the precipitate in 1.0 ml chloroform. Ag nanocube monolayers were fabricated using a KSV Nima KN2001 Langmuir–Blodgett trough, as previously described^17^. The Ag nanocube solution was deposited dropwise onto a deionized water (18 Mω) subphase. The nanocube film formed at the air–water interface was allowed to sit for 30 min after deposition to allow the interfacial surface pressure to reach 0.0 mN m^−1^. This insures complete solvent evaporation, enables excess PVP desorption into the water subphase, and allows nanocubes to disperse isotropically at the interface. The Ag nanocube film was isothermally compressed to surface pressures ranging from 0 to 50 mN m^−1^ before transfer onto a solid substrate. This transfer was carried out using a vertical dipper arm that is drawn through the air–water interface at a speed of 0.5 mm min^−1^.

### Substrate preparation

Substrates for nanocube deposition were fabricated by first cleaning a soda-lime glass wafer (University Wafers) with a piranha solution (3:1 mixture of concentrated sulphuric acid and hydrogen peroxide 30% vol vol^−1^) and plasma treatment (Harrick Plasma Cleaner PDC-002) for 300 s at 30 W. The substrates were then put into a high vacuum sputter chamber (Denton Discovery 18 Sputter System), and cleaned for 60 s with a 100 W RF Ar plasma. The substrates were then sputtered with a 5 nm Ti adhesion layer, followed by a thin Au layer ranging from 15to 75 nm. To generate a spacer layer between the Ag nanocubes and the Au thin film, a dielectric layer atop the Au thin film is necessary. To fabricate dielectric layers with variable thicknesses, we employed two general methods: (i) formation of a self-assembled monolayer (SAM) of alkanethiols on the Au thin film to give a spacer layer on the order of a few nanometres; and (ii) a spin-coated polymer thin film was used to provide a dielectric layer with a thickness between 15 and 100 nm. The SAMs were fabricated using carboxyl-terminated alkanethiols with varying length alkyl chains: 6-Mercaptohexanoic acid; 11-Mercaptoundecanoic acid; and 16-Mercaptohexadecanoic acid for dielectric layers that are approximately 0.89, 1.39 and 2.01 nm, respectively (Rudolph Auto EL Ellipsometer). The sputtered glass substrates were incubated in a 5.0 mM ethanolic solution of the desired alkanethiol for 60 min, followed by sonication in acetone for 15 min and oxygen-plasma treatment for 15 min to remove any excess physisorbed molecules. To fabricate the polymer layers, a 0.5–2.0 wt% solution of poly(methyl methacrylate) (PMMA; *M*_w_=25,000) in toluene was spin coated (Laurell WS 400BZ-6NPP/lite) onto the sputtered glass substrates at different speeds depending on the desired thickness. The thickness of the SAMs and PMMA films were verified by ellipsometry.

### Optical measurements

All transmittance and reflectance spectra were obtained using a Perkin-Elmer Lambda1050 UV-Vis-NIR Spectrometer. A piranha cleaned soda-lime glass wafer was used to obtain a background spectrum for the 100% transmittance reference beam. All specular reflectance measurements were taken at 4° near-normal incidence. Transmittance and reflectance spectra of the Ag nanocube/Au thin films were compared with a reference spectrum of an appropriately thick Au thin film on glass. For all reflectance and transmittance measurements, a spot size of ∼20 mm^2^ was used, covering over 50% of the fabricated surface (excluding a ∼1 mm perimeter around the edge of each substrate). This is to ensure effective measurement of the ensemble nanostructured metasurface and to provide an average over regions of variable uniformity. Absorption spectra were obtained by subtracting per cent reflection and transmission from 100%.

### FDTD Simulations

Finite difference-time domain simulations were carried out using the commercially available Lumerical FDTD Solutions utilizing the triton shared-computing cluster (TSCC) at the University of California, San Diego. Two-dimensional FDTD modelling is used to determine the nature of the electric and magnetic field confinement at the nanocube's surface and its interaction with the adjacent metallic film. Due to the model's 2D geometry the higher order modes of the silver cube are skewed, because they tend to involve multiple faces of the cube simultaneously. This model does not take into account quantum effects, which are typically observed when the geometry of the simulations start to approach dimensions of ∼2 nm. A 2D model was used to cut down the computational costs and due to the ease of implementing periodic boundary conditions.

## Additional Information

**How to cite this article**: Rozin, M.J. *et al.* Colloidal metasurfaces displaying near-ideal and tunable light absorbance in the infrared. *Nat. Commun.* 6:7325 doi: 10.1038/ncomms8325 (2015).

## Supplementary Material

Supplementary Information Supplementary Figures 1-12, Supplementary Tables 1-2, Supplementary Note 1 and Supplementary References
