# Supplementary material for: Colloidal metasurfaces displaying near-ideal and tunable light absorbance in the infrared
Source: Nat Commun. 2015 Jun 23;6:7325. doi: 10.1038/ncomms8325 (PMC4557363; doi:10.1038/ncomms8325)
Supplement: Supplementary Information — Supplementary Figures 1-12, Supplementary Tables 1-2, Supplementary Note 1 and Supplementary References [file ncomms8325-s1.pdf]

Supplementary Information

Supplementary Figures

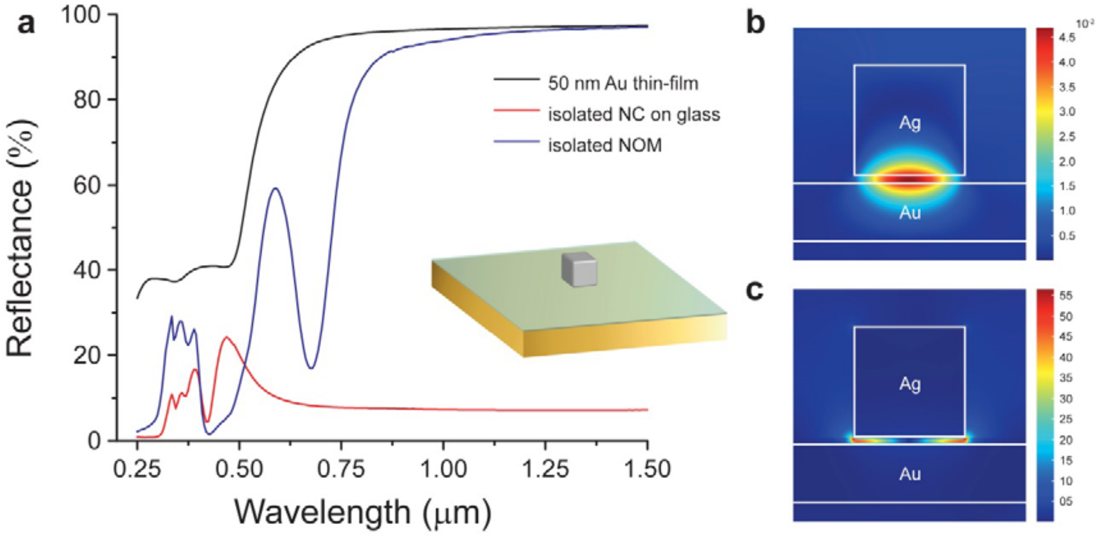

Supplementary Figure 1 | **Electromagnetic modes in isolated nanocube-on-metal metasurfaces.** (a) Experimental reflectance spectra for an array of isolated Ag nanocubes (*e*=92 nm, *d*>300 nm) on glass (red curve), and on a 50 nm Au thin-film (blue curve), showing the significant redshift of the dipolar nanocube resonance. For reference, the reflectance for a 50 nm Au film is also plotted (black curve). For λ<425 nm, the spectral features associated with the higher-order nanocube LSPRs, there is virtually no frequency shift of these modes with the addition of the Au thin-film. For wavelengths beyond the gap-mode (λ>850 nm), the reflectance mirrors that of the underlying Au film. FDTD simulation of the (b) magnetic and (c) electric field density at the gap-mode resonance shows that the enhanced field is confined within the cavity generated by the nanocube.

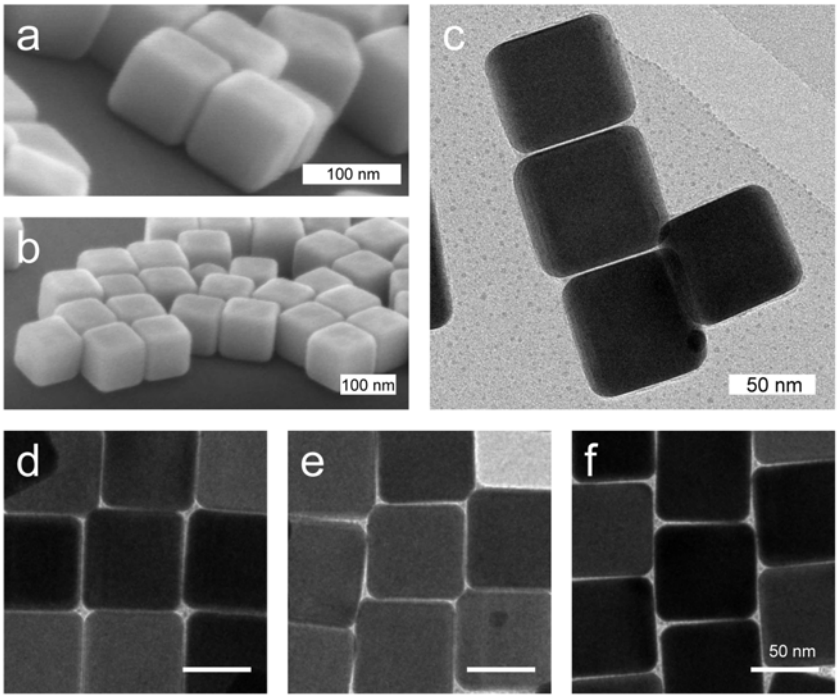

Supplementary Figure 2 | **Interparticle spacing of close-packed Ag nanocubes.** (a,b) SEM and (c-f) TEM images of close-packing Ag nanocubes capped with 55k M_w PVP. Nanocubes at the air-water interface have PVP chains that extend out radially for surface pressures at or near 0 mN m^{-1}. At surface pressures above ~2 mN m^{-1}, steric interactions between the polymer chains of interacting nanocubes is no longer elastic. The nanocubes interact with each other through van der Waals interactions, compressing the polymer chains between nanocube faces, creating a uniform dielectric shell around each cube. The average gap distance between nanocube faces was measured at 1.73±0.43 nm.

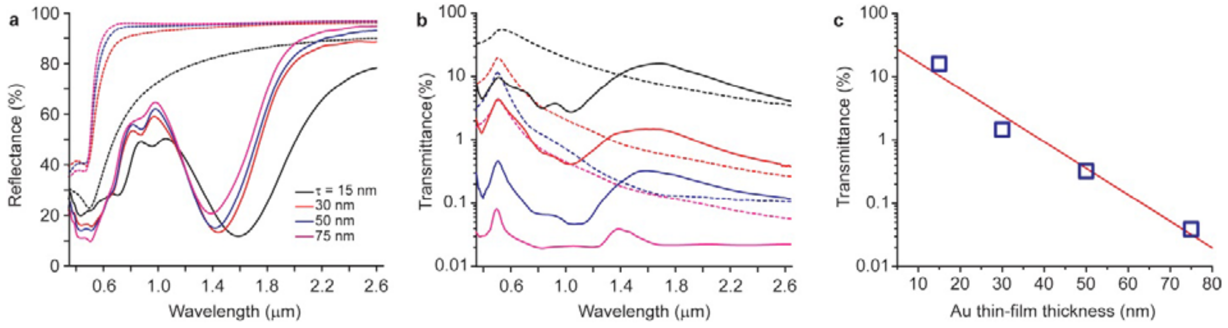

Supplementary Figure 3 | **Effect of Au thin-film thickness on NOM fundamental resonance**. (a,b) Near-normal reflectance and transmittance spectra for close-packed NOM structures with 70 nm Ag nanocubes and Au thin-film thickness of τ=15, 30, 50, and 75 nm. Solid lines show the optical response of the aforementioned NOM metasurfaces of varying τ, whereas dashed lines show the response for bare Au thin-films of corresponding thickness. (c) The maximum transmission at the fundamental mode of each metasurface is plotted in a semi-log fashion, where a linear increase in the Au thin-film thickness will result in a nearly exponential decrease in NOM transmittance at the resonant frequency.

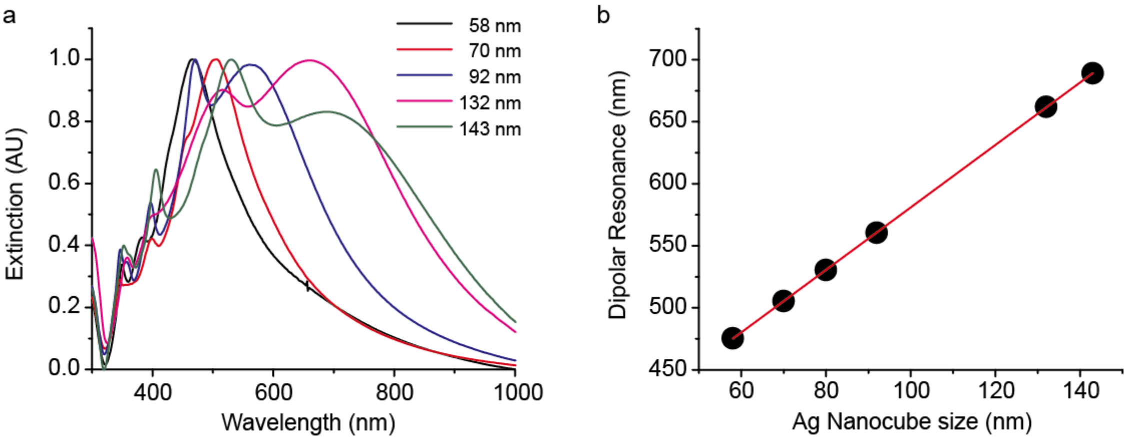

Supplementary Figure 4 | **Linear trend of Ag nanocube dipolar resonance frequency with nanocube size**. (a) Normalized extinction spectra of colloidal Ag nanocubes with various edge lengths (*e*=58.5±3.6, 69.8±3.8, 92.3±5.1, 132.1±6.6, and 143.4±5.9 nm). (b) Trend showing the linear relationship between the dipolar LSPR wavelength of the nanocubes versus nanocube size, measured as the average nanocube edge length.

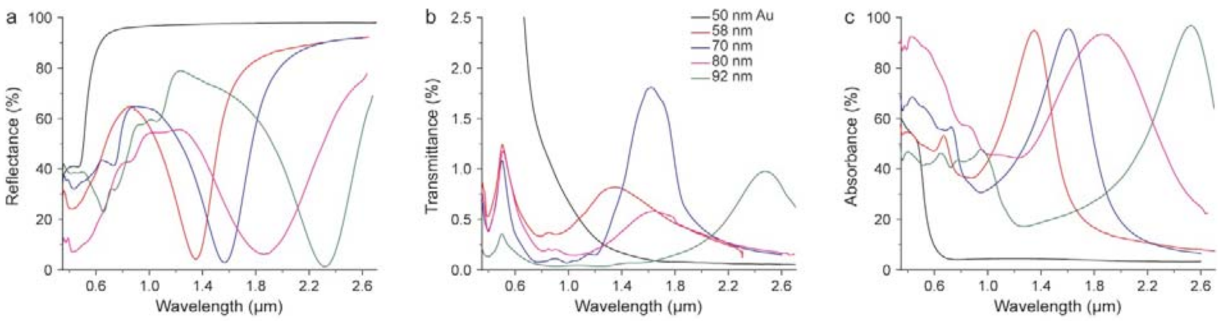

Supplementary Figure 5 | **Optical response of NOM metasurfaces fabricated with nanocubes of varying size**. (a) Near-normal reflectance, (b) transmittance, and (c) calculated absorbance spectra for metasurfaces with nanocubes of different edge lengths (*e*=58.5±3.6, 69.8±3.8, 80.2±7.9, and 92.3±5.1 nm).

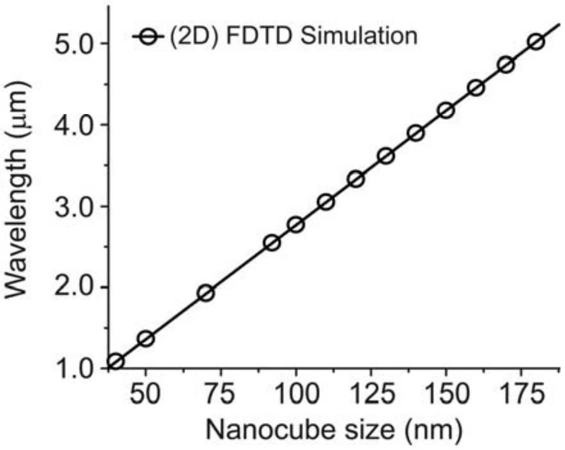

Supplementary Figure 6 | **Metasurface Fundamental Mode beyond the Near-IR**. 2D FDTD Simulations of close-packed NOM metasurfaces with varying nanocube size from *e* = 40-200 nm. In order to extend the perfect-absorbing fundamental mode out to λ=3.0, 4.0, and 5.0 μm, NOM metasurfaces would require nanocubes with edge lengths e=108.1 nm, 143.7 nm, and 179.3 nm, respectively.

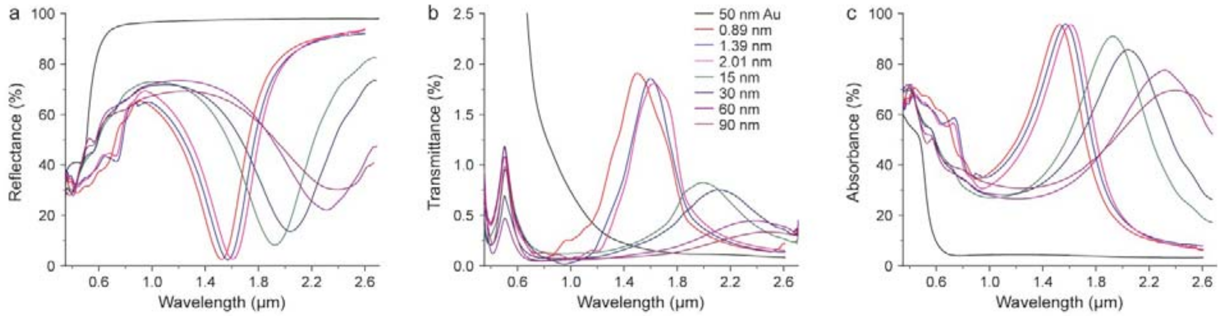

Supplementary Figure 7 | **Optical response of NOM metasurfaces fabricated with dielectric spacer layer of variable height**. (a) Near-normal reflectance, (b) transmittance, and (c) calculated absorbance spectra for close-packed NOM metasurfaces fabricated with 70 nm Ag nanocubes, with two regimes of variable dielectric spacer height. The first height regime used a self-assembled monolayer of one of three alkane-thiol monomers of variable chain length, with dielectric constant n=1.49. This enabled spacer heights within the extreme near-field coupling regime (h≈2.6, 3.1, and 3.7 nm). The second regime employed spin-cast PMMA layers (n=1.52) to achieve spacer heights within the strong-to-weak near-field coupling regime (h≈15, 30, 50, and 75 nm).

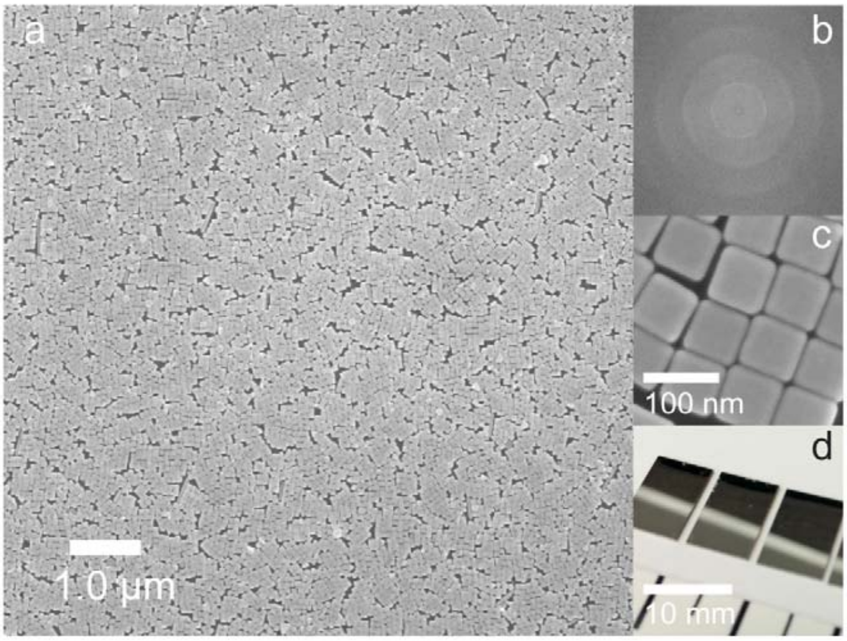

Supplementary Figure 8 | **Metasurface long-range order**. (a) SEM image of long-range order of Ag nanocube metasurface. (b) FFT power spectrum of 10x10 µm area, showing uniform average spacing. (c) Close-up of nanocube film order, showing close-packed face-to-face nanocubes. (d) Digital color image of as-made metasurfaces, fabricated on 10x7.5 mm substrates.

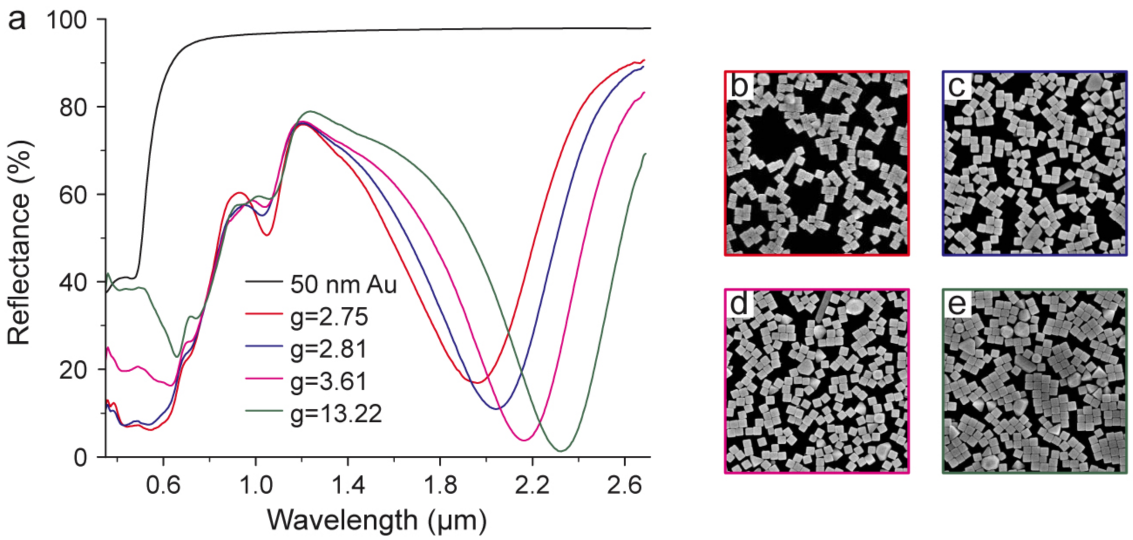

Supplementary Figure 9 | **Effect of nanocube arrangement & domain size on the NOM metasurfaces optical response**. (a) Near-normal reflectance and the corresponding (b-e) SEM images for metasurfaces with varying domain sizes of *g*= 2.75±1.80, 2.81±1.87, 3.6±1.41, and 13.22±4.76 nanocubes. The NOM structure used 92 nm Ag nanocubes supported on a 50 nm Au thin-film by a 3 nm dielectric spacer height.

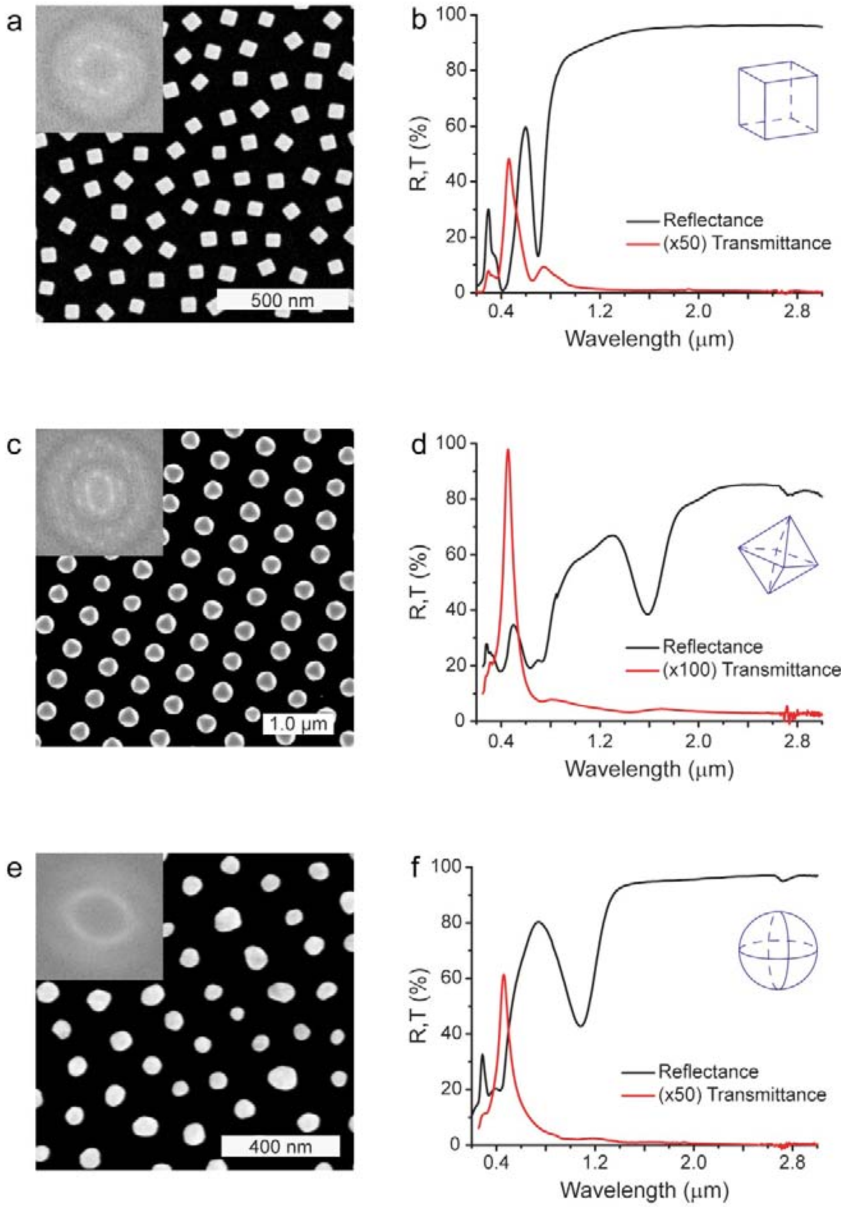

Supplementary Figure 10 | **Nanocrystal shape dependence for low-density metasurfaces**. SEM images corresponding to near-normal reflectance and transmittance spectra for low-density metasurfaces made with (a,b) Ag nanocubes, (c,d) Ag octahedra, and (e,f) Ag spheroids. Inset shows each nanocrystal array's FFT analysis, with greatest uniformity in nanocrystal shape and spacing for the nanocube film, and decreasing as the particles deviate from having cubic symmetry. Structural, optical, and quality parameters are listed in Supplementary Table 2.

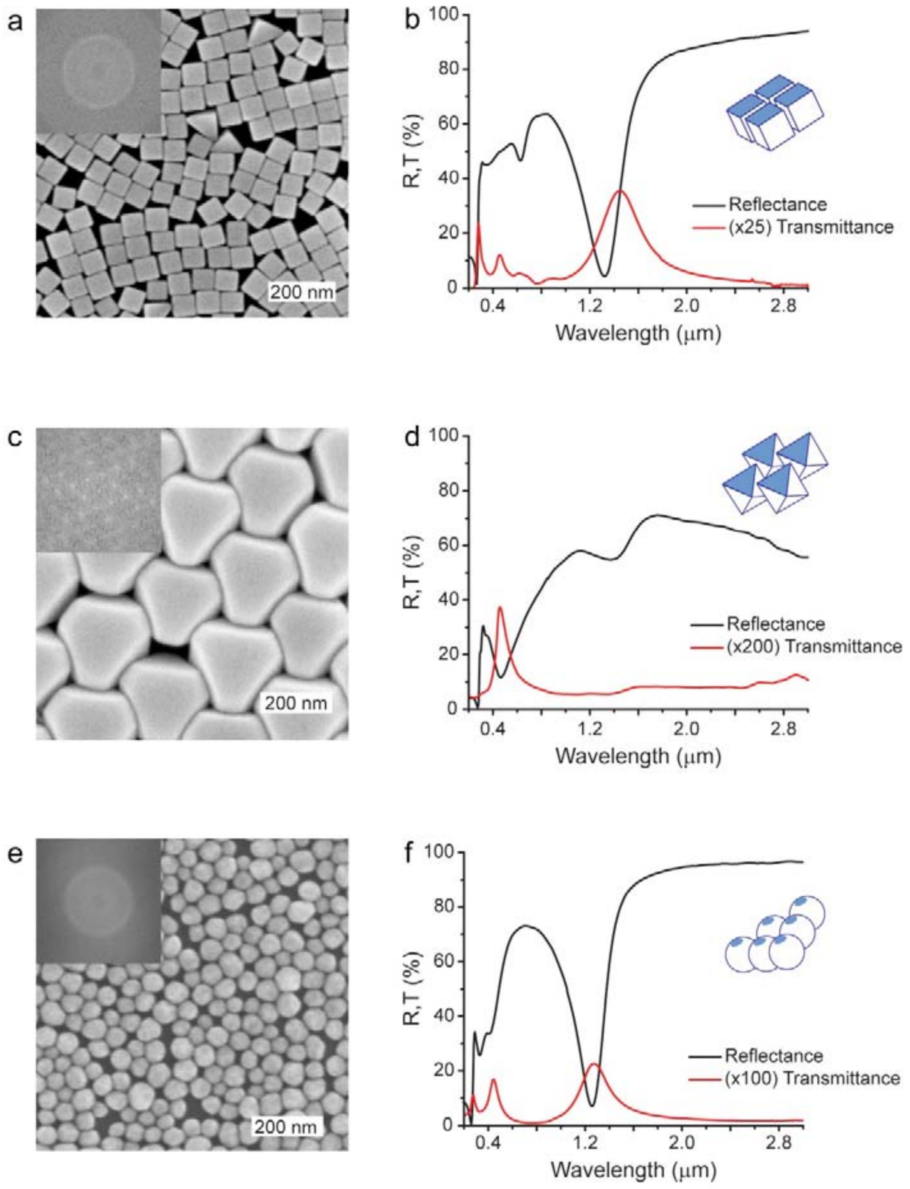

Supplementary Figure 11 | **Nanocrystal shape dependence for close-packed metasurfaces**. SEM images corresponding to near-normal reflectance and transmittance spectra for close-packed metasurfaces made with (a,b) Ag nanocubes, (c,d) Ag octahedra, and (e,f) Ag spheroids. Inset shows each nanocrystal array's FFT analysis. The octahedra, which close-pack to form an interlocking array, produce a well-ordered hexagonal lattice, as denoted by the spot pattern of that sample's FFT. The cubes and spheroids, which show larger size dispersity, form a random monolayer. The circular ring in each sample's FFT image denotes a monolayer where individual particles are uniformly spaced but randomly oriented, with the sharper (narrower line width) ring in the nanocube array's FFT coming from the higher degree of uniformity of the nanocube particles.

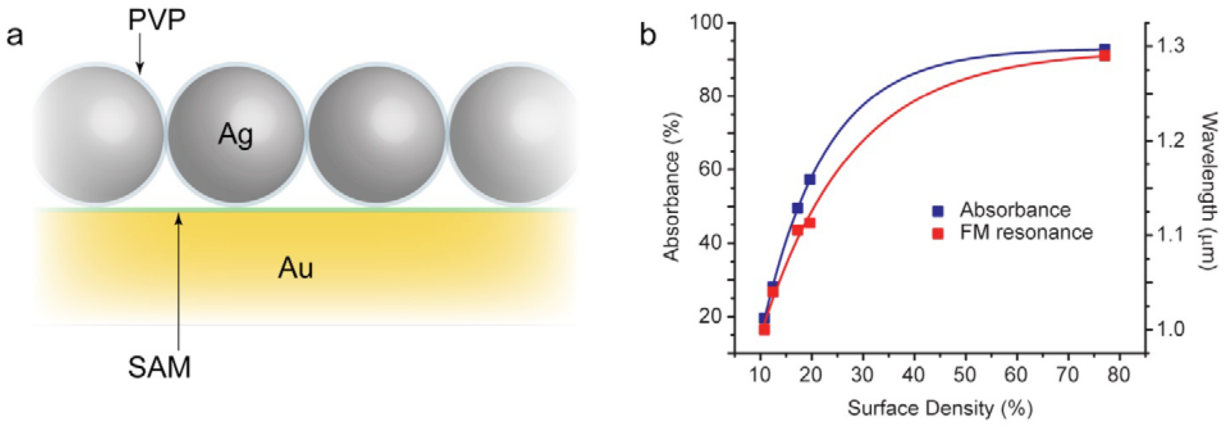

Supplementary Figure 12 | **Close-packed metasurface fabricated with Ag nanospheres of varying surface densities**. (a) Schematic of Ag nanosphere-on-metal metasurface, showing the reduced interaction-footprint of the particle on the underlying thin-film, as compared to a nanocube or octahedra. Metasurfaces with 65 nm Ag spheroids of varying surface density atop a 50 nm Au thin-film were fabricated. (b) Trend showing the position of the fundamental resonance, as well as the percent absorbance at that resonance vs. particle surface density. The cavity produced by two adjacent spheroids results in minimal inter-particle coupling, resulting in a direct correlation between position of the fundamental gap-mode, and percent absorbance at the gap-mode, for metasurfaces of varying surface density.

e (nm)	%SC	h (nm)	λ_R (µm)	FWHM (µm)	%R (λ_R)	%T (λ_R)	%A (λ_R)
58.5 ± 3.6	72.0	~3	1.350	0.3434	4.25	0.82	92.89
69.8 ± 3.8	71.1	~3	1.566	0.4245	2.86	1.81	90.98
80.2 ± 7.9	78.4	~3	1.865	0.7810	6.16	0.58	91.71
92.3 ± 5.1	85.3	~3	2.557	0.4120	2.13	0.98	97.87

Supplementary Table 1 | **Structural and optical parameters for metasurfaces with various nanocube size.** Various structural and optical parameters for the metasurfaces in Supplementary Fig. 5. The average nanocube edge length (e) is varied for metasurfaces fabricated with approximately close-packed nanocube films, meaning the percent surface coverage (%SC) of nanocubes on the Au layer greater than 70%. The variation in spectral lineshape is primarily attributed to heterogeneities in size and shape within each colloidal nanocube batch.

shape	packing	*e* (nm)	%SC	*d* (nm)	λ_r (μm)	FWHM (μm)	Q_f	%R (λ_r)	%T (λ_r)	%A (λ_r)
cubes	isolated	58.5 ± 3.6	21.0	133.9 ± 5.9	0.735	0.1016	7.2	13.10	0.18	86.77
	close-packed	58.5 ± 3.6	81.9	63.3 ± 1.3	1.350	0.3434	3.9	4.25	1.42	94.75
octahedra	isolated	235.3 ± 12.1	22.2	477.8 ± 97.8	1.610	0.2622	6.1	38.37	0.04	61.59
	close-packed	235.3 ± 12.1	88.0	247.9 ± 5.5	-	-	-	-	-	-
spheroids	isolated	65.3 ± 10.8	20.1	134.8 ± 19.8	1.112	0.2890	3.8	42.70	0.05	57.26
	close-packed	57.2 ± 5.6	77.1	60.3 ± 0.6	1.290	0.2725	4.7	7.10	0.23	92.67

Supplementary Table 2 | **Structural, optical, and quality parameters for metasurfaces with various shaped nanocrystals.** Various measured parameters for low-density and close-packed metasurfaces made with Ag nanocubes, Ag octahedra, and Ag spheroids, corresponding to the metasurfaces in Supplementary Figures 10 and 11. Due to the relative large size of the Ag octahedra, close-packed metasurfaces made these particles produce a fundamental resonance beyond 3.3 μm, the maximum range of our spectrometer.

Supplementary Note 1: Estimation of Mode Volume

We estimate mode volume using statistical image analysis to examine transmission electron microscope (TEM) images of Ag nanocube arrays. We selected only nanocubes oriented with a face precisely normal to the electron beam, as to mitigate error from the increased cross-sectional area of a plane not parallel to a nanocube facet. We measured the face-to-face edge lengths (e_1, e_2) and the radius of curvature of nanocube edges (r_e), and calculated the resulting cross-sectional area (A_{gap}). Multiplying this area by the height of the gap between the nanocube and the metallic film gives an approximation of the local field volume for a NOM metasurface with isolated nanocubes. The gap height (h) is the sum of the self-assembled alkanethiol monolayer height (as measured by ellipsometry) and the thickness of the compressed polymer layer encapsulating the nanocube (measured via TEM as one half the spacing between two close-packed nanocube faces).

$$A_{gap} = (e_1 \bullet e_2) - r_e{}^2[4 - \pi] \qquad V_{gap} = A_{gap}\left[2.10\,nm + \frac{1}{2}(1.73\,nm)\right]$$

To determine the relative change in local field volume as we transition from the isolated to the close packed NOM metasurface, we simply adapt the above method for TEM images of close packed Ag nanocubes. The volume between abutting nanocube faces in the close packed film was calculated by selecting a region of the film where all neighboring nanocubes lay in a single plane, which was oriented precisely normal to the incident electron beam (for a selection of images used in the analysis, see supplementary figure 2, d-f). In this manner, all of the inter-nanocube spaces in the region of interest are approximately orthogonal to the image plane. We then measured the cross-sectional area of the void space surrounding a selected nanocube (A_{IP}). Most of void space is formed between two approximately parallel nanocube faces, with average spacing of ~2 nm, governed by the chain length of the PVP coating. However where the nanocubes are offset, leading to a larger volume void extending away from the nanocube face, we considered the void region extending ~5 nm beyond the perimeter of the selected nanocube. This distance was chosen because it is on the order of the LSPR decay length for strongly interacting particles.[1] We then approximate the inter-particle local field volume by multiplying the cross-sectional area of this void space by the height of the nanocube, calculated from the average of e_1 and e_2. The sum of the inter-particle volume and the nanocube-metal film gap yields the approximate volume of the local field for a close packed nanocube.

$$V_{close-packed} = \frac{A_{IP}}{2}(e_1 + e_2) + V_{gap}$$

This leads to an average local field volume for nanocubes (average edge length e=74.98±1.79 nm) in the close-packed NOM metasurface of approximately 2.77±0.23 times that for the metasurface with isolated nanocubes.

REFERENCES

1. Atay, T., Song, J.-H. & Nurmikko, A.V. Strongly Interacting Plasmon Nanoparticle Pairs: From Dipole−Dipole Interaction to Conductively Coupled Regime. *Nano Letters* **4**, 1627-1631 (2004).
